# Evaluation of the Heshang Cave stalagmite calcium isotope composition as a paleohydrologic proxy by comparison with the instrumental precipitation record

**DOI:** 10.1038/s41598-018-20776-5

**Published:** 2018-02-08

**Authors:** Xiuli Li, Xueping Cui, Dong He, Jin Liao, Chaoyong Hu

**Affiliations:** 1 State Key Laboratory of Biogeology and Environmental Geology, China University of Geosciences, Wuhan, 430074 China; 2 Department of Geography, School of Earth Sciences, China University of Geosciences, Wuhan, 430074 China

## Abstract

With their merits of precise dating and sensitivity to climate changes, laminated stalagmites are an important terrestrial archive for reconstructions of paleohydrological changes. In particular, the Ca isotope composition (δ^44/42^Ca) of the Heshang Cave stalagmite has been documented to record a precipitation decrease during the 8.2 ka event in central China. As an extension, this study directly compares near-annual resolution δ^44/42^Ca data with an instrumental precipitation record to evaluate the fidelity of δ^44/42^Ca as a paleohydrologic proxy on annual to decade timescales. Over the period 1881–2001 AD, the δ^44/42^Ca values correlate significantly with both precipitation from a nearby weather station and the dryness/wetness index in the middle Yangtze River, with a stronger correlation on decadal smoothed data. These results clearly show that the δ^44/42^Ca ratio from stalagmites is an effective proxy for paleohydrological changes on a decadal timescale. More study is encouraged to refine understanding of stalagmite Ca isotope ratios and hydrological conditions and their application in paleohydrologic reconstructions.

## Introduction

In the monsoon region of East Asia, drought events caused by decreased monsoon rain intensity often bring severe disasters^1,2^. According to China’s third assessment report on climate changes^3^, an increase appears both in the area influenced by droughts and in the frequency of severe droughts from 1951 to 2010. In order to effectively understand the mechanisms responsible for drought disasters, reconstruction of high resolution records of past extreme drought events is needed beyond the timescale of instrument records, which is usually ca. 50–100 yr.

Stalagmites with laminated layers present an excellent terrestrial archive to reconstruct paleohydrologic histories in the monsoon regions, and they can be easily dated by layer counting^4–6^. Over the last few decades, numerous proxies have been developed through study of stalagmites, for example, calcite oxygen isotope ratios^6,7^, calcite carbon isotope ratios^4,8^, trace elements^9,10^, and carbon isotope ratios of organic matter^11^. The calcite oxygen isotope ratios (δ^18^O) are widely applied to track monsoon intensity and associated hydrological changes in the late Quaternary^6,7,12^. In eastern China, the stalagmite δ^18^O ratio mainly reflects circulation changes or monsoon changes^13^. The ratios of trace elements such as Mg, Sr, Ba relative to Ca have also been widely used to qualitatively reflect changes of rainfall amounts^10^. However, these proxies, which are affected by several factors, have not been used to quantitatively reconstruct drought events and to estimate the extent of drought.

In a recent study, Owen *et al*.^14^ proposed that stalagmite Ca isotope compositions (δ^44/42^Ca) have the potential to be used as a proxy for aridity. In cave settings, calcium preserved in stalagmites mainly comes from bedrock carbonate and is transported by groundwater containing abundant HCO_3_^−^. When groundwater infiltrates through unsaturated zones, secondary carbonate will precipitate in aquifers, fractures, and cave ceilings in a process known as prior calcite precipitation (PCP)^15^. Such a process is caused by degassing in the unsaturated zone. Previous studies have proposed that PCP varies closely with water-residence time and associated drought^16,17^. Paralleling PCP, Ca isotopes fractionate between the secondary calcite and Ca ion remaining in solute^14,18,19^. Through the study of the δ^44/42^Ca values in modern dripwater, glass plate calcite, and the dolomite bedrock collected from Heshang Cave, central China, Owen *et al*.^14^ argued that PCP was the major process that controls the variations of stalagmite δ^44/42^Ca values. Enhanced PCP will result in a positive excursion of the Ca isotope composition ultimately preserved in stalagmites^14^. Aided by previous studies that proposed the extent of PCP correlates with local effective rainfall^20,21^ and the knowledge of Ca isotope fractionation in modern regimes, Owen *et al*.^14^ semi-quantitatively estimated that precipitation was reduced by one-third relative to the present during the onset of the 8.2 ka event.

In the study of Owen *et al*.^14^, only a one-point calibration between δ^44/42^Ca and precipitation was utilized. In order to confirm the reliability of stalagmite Ca isotope ratios as a paleohydrologic proxy, it is worth extending the previous work by comparing the near-annual resolution Ca isotope record with the local instrumental precipitation record and to test the potential of the Ca isotopes to record severe drought events on an annual timescale. Here we provided an annual resolution Ca isotope record from Heshang Cave, the same site studied by Owen *et al*.^14^ for the period 1881–2001 AD with the goal of evaluating its relationship with the local instrumental precipitation record.

## Materials and Methods

For this study, stalagmite samples were collected from the Heshang Cave (30°27′N, 110°25′E, 294 m altitude). This cave is located on the south bank of the Qingjiang River, central China. For more detailed information on the Heshang Cave, please refer to Hu *et al*.^22^. Generally, climate in this site has a distinct seasonality, with a warm-wet summer and a cool-dry winter. The mean annual temperature is 18 °C, while mean annual precipitation is 1144 mm.

Samples were obtained from the top of the HS6 stalagmite along the growth axis. HS6 is a laminated stalagmite that was cut from the cave in 2004^22^. Its chronology is based on layer counting^23^, referring to the top as 2004. Close agreement between layer counting and U/Th dates confirmed the laminated layers to be annual layers^6,23^. As a consequence of the studies of He *et al*.^23^ and Liao and Hu^24^, samples from some layers were exhausted. In this study, we were able to select samples for the period from 1881–2001 AD for elemental and calcite isotope analyses.

### Trace element analysis

The trace element analysis was conducted on a Thermo IRIS Intrepid II XSP Inductively coupled plasma atomic emission spectroscopy (ICP-OES) at the State Key Laboratory of Biogeology and Environmental Geology, China University of Geosciences. Mg/Ca, Sr/Ca, and Ba/Ca were analyzed using the “ratio” method. Samples were dissolved in 2% double distilled HNO_3_ that was also used as the blank solution and to dilute standards. The concentrations of these elements were computed from a standard curve and expressed as element/Ca ratios.

### Calcium isotope analysis

**Figure 1:**
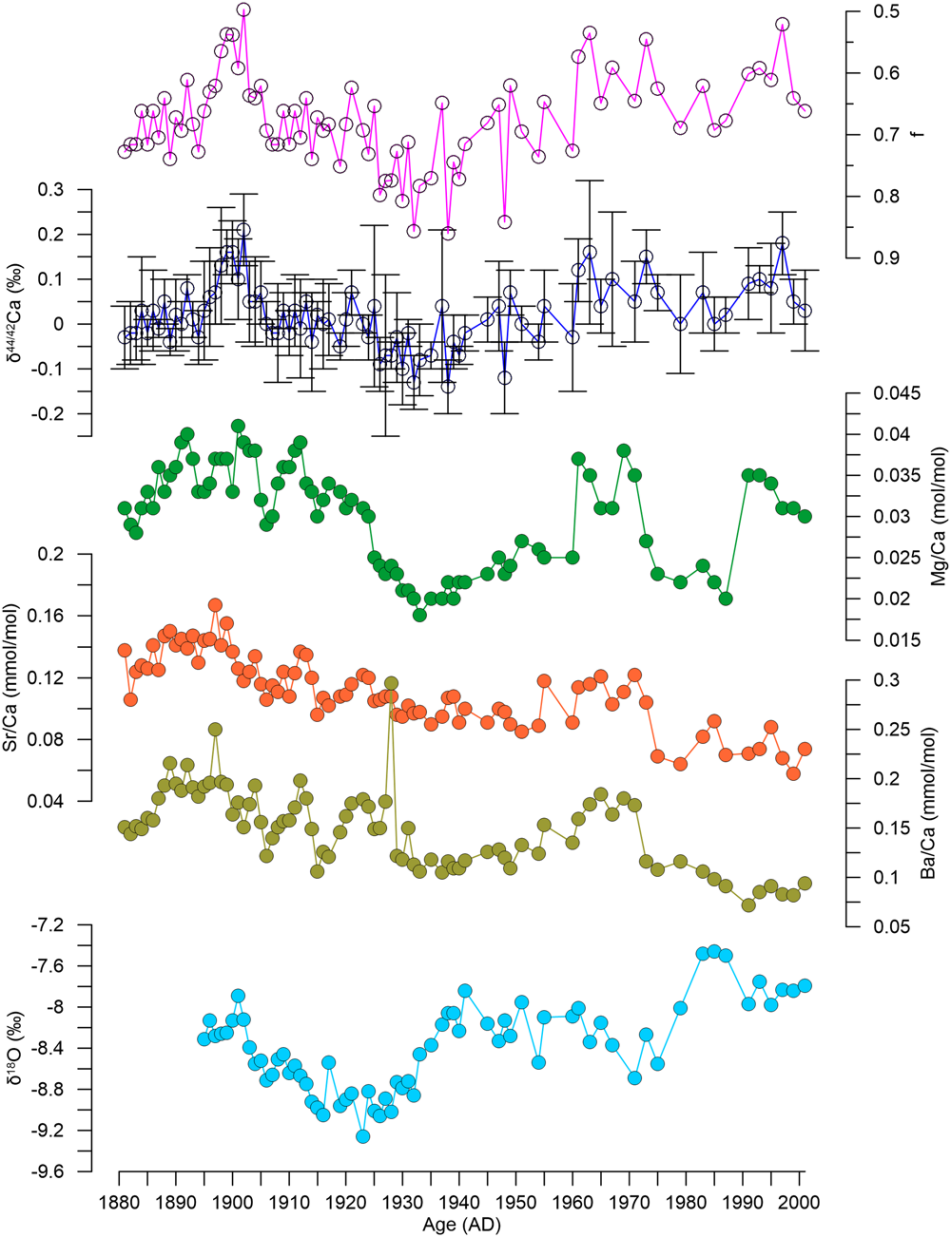
Time series of δ^44/42^Ca values and Sr/Ca, Mg/Ca, Ba/Ca, and δ^18^O ratios, and the calculated f values in the HS6 samples spanning 1881–2001 AD.

The procedures for separation and analysis of Ca isotope compositions followed the approach used in Owen *et al*.^14^. In the prior study of Ca isotope composition in Heshang Cave, Owen *et al*.^14^ documented that no processing was necessary for speleothem samples owing to the quite low trace element contents. In the HS6 samples, the relative contents of Sr and Mg (Sr/Ca = 0.13 mmol/mol; Mg/Ca = 36.8 mmol/mol) were also quite low (Fig. 1). Thus, the column chemistry steps were omitted in this study.

Ca isotope analysis was performed on a Nu plasma II Instruments MC-ICP-MS with Aridus desolvating nebuliser at China University of Geosciences, following the approach used in Owen *et al*.^14^. Samples were standardized relative to NIST SRM 915a utilizing a sample-standard bracket method. Each sample was analyzed more than 12 times. A second standard (NIST SRM 915b) and a secondary internal standard (HPS Ca), identical with Owen *et al*.^14^, were used during the Ca isotope analysis. We used the method of Owen *et al*.^14^ to correct Sr interference.

The Ca isotope compositions were expressed as δ^44/42^Ca values. For NIST SRM 915b, the measured calcium isotope value was δ^44/42^Ca = 0.36 ± 0.08‰ (2**σ**, n = 42), and for HPS, the measured calcium isotope value was δ^44/42^Ca = 0.25 ± 0.07‰ (2**σ**, n = 38) in our laboratory. The above measured values agree well with those reported in Owen *et al*.^14^. In addition, a calcite sample from the HS4 stalagmite from Heshang Cave was used as our lab internal standard (named STM), with a mean value of δ^44/42^Ca = 0.31 ± 0.07‰ (2**σ**, n = 40).

## Results

**Figure 2:**
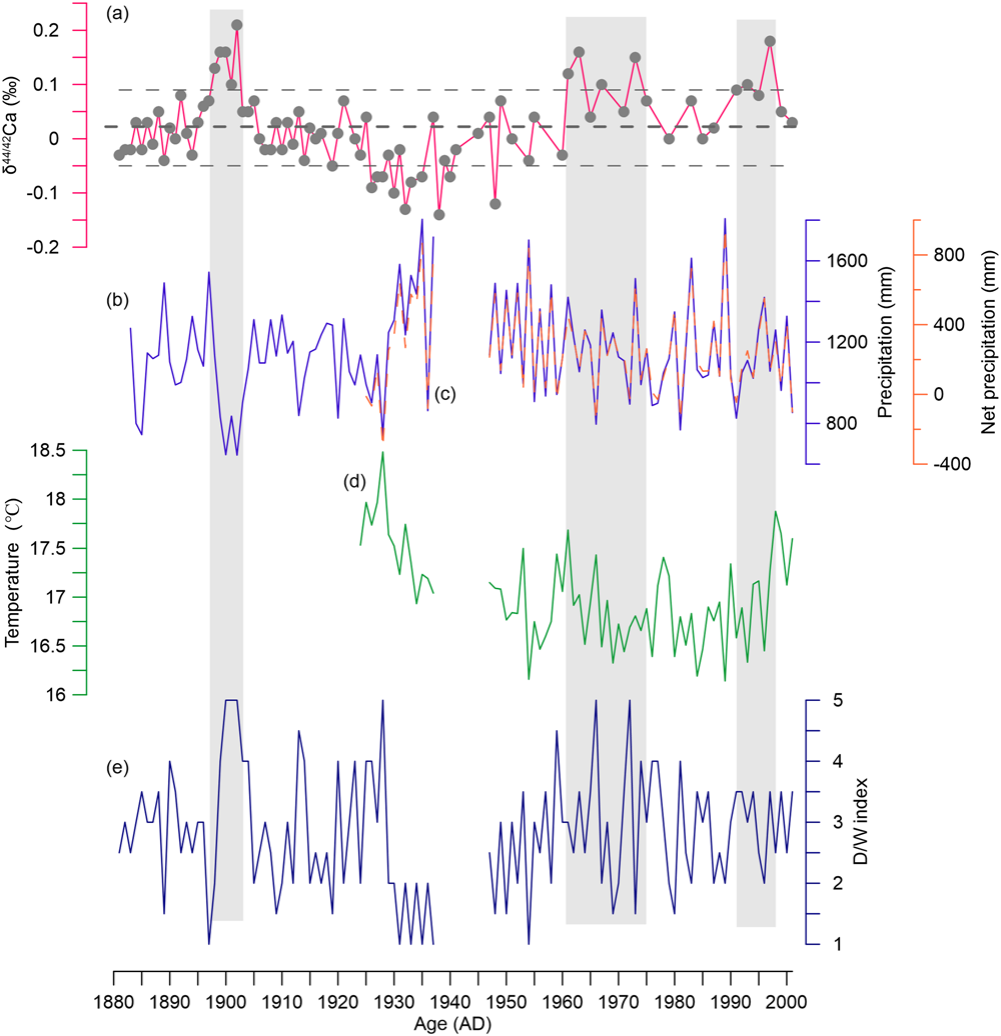
Comparisons of the δ^44/42^Ca values (**a**) with the yearly precipitation (**b**), net precipitation (**c**), yearly air temperature (**d**) in Yichang, and the averaged dryness/wetness index from Wuhan and Jiangling^27^ (**e**). The dryness/wetness index in a year is classified into 5 grades: grade 1- very wet, grade 2- wet, grade 3- normal, grade 4-dry, and grade 5-very dry. The dashed horizontal lines in (a) refer the upper limit and lower limit of deviation from the averaged δ^44/42^Ca value (bold line) during 1881–2001.

For the period 1881–2001 AD, the δ^44/42^Ca values varied between −0.14‰ and 0.21‰ and averaged 0.02‰ (Fig. 1). In this 121-yr period, three intervals have markedly larger δ^44/42^Ca values: 1896–1905 AD (*t*-test, *p* < 0.001), 1961–1975 AD (*t*-test, *p* < 0.01), and 1991–1999 AD (*t*-test, *p* < 0.05; Fig. 1). During the 1990s, the positive excursion of δ^44/42^Ca values in most data points is undistinguishable with the instrumental error (0.07‰; Fig. 2). The elemental ratios of Mg/Ca, Sr/Ca and Ba/Ca have higher values over a wide interval in the early 1900s (Fig. 1). Both Mg/Ca and Sr/Ca ratios clearly increase from 1961–1975 AD, while the Ba/Ca ratio has only a moderate increase. From 1991–2001 AD, only the Mg/Ca ratio parallels the increase of δ^44/42^Ca values. Fluctuations of the δ^18^O ratio show three distinctive stages, with less negative values in 1897–1906 AD, more negative values in 1903–1930 AD, and then less negative values upwards (Fig. 1).

## Discussion

### Comparing δ^44/42^Ca in HS6 with instrumental precipitation

In the previous work on the cave Ca isotope ratios, Owen *et al*.^14^ provided robust evidence to support the hypothesis that cave δ^44/42^Ca ratio was a novel proxy for drought. This study can further support this hypothesis by comparing the near annual resolution Ca isotope records directly with an instrumental precipitation record (Fig. 2). The annual precipitation data were obtained from the Yichang Meteorological Station. The data between 1938–1946 were absent because of the Japan invasion into China during the Second World War. The calculation of net precipitation followed Willmott *et al*.^25^ and Tremaine and Froelich^26^. Historical documentary data can also be cited to evaluate the potential of the speleothem δ^44/42^Ca ratio as an index of drought on the annual timescale. Here we use the mean dryness/wetness index (D/W) from nearby Jiangling and Wuhan from the benchmark publication of “Atlas of droughts and floods for the last 500 years”^27^. All these cities are located in the middle reaches of the Yangtze River Basin and have a similar climate context as the Heshang Cave. The δ^44/42^Ca values correlate negatively with the annual rainfall, the summer precipitation, and positively with the averaged D/W index (Table 1). The seemingly positive relation between δ^44/42^Ca and yearly air temperature probably results from the close relation between air temperature and net precipitation. Therefore, the results of this study clearly support the hypothesis that the Ca isotope ratio can indicate aridity. The not very strong correlation between δ^44/42^Ca values and rainfall indicates that other factors can mediate the isotope fractionation between solute and speleothem, such as calcite growth rate^18^.

**Table 1:** Correlation analysis for Ca isotope values with precipitation, net precipitation, air temperature and the D/W index (^**^p < 0.01; ^*^p < 0.05).

	δ^44/42^Ca	P_3–5_	P_6–8_	P_9–11_	P_12–2_	P_total_	P_eff_	Temp
P_3–5_^a^	−0.15							
P_6–8_^b^	−0.34^**^	0.09						
P_9–11_^c^	0.09	−0.06	0.09					
P_12–2_^d^	−0.15	0.07	−0.05	0.02				
P_tot_^e^	−0.29^*^	0.36^**^	0.83^**^	0.51^**^	0.14			
P_net_^f^	−0.07	0.27^*^	0.84^**^	0.55^**^	0.14	0.99^**^		
Temp^g^	−0.44^**^	−0.02	−0.12	−0.31^**^	−0.08	−0.25^*^	−0.37^**^	
D/W	0.34^**^	−0.27^**^	−0.78^**^	−0.31^**^	−0.04	−0.83^**^	−0.83^**^	0.23

^a^Spring (March-May) precipitation; ^b^summer (June-August) precipitation; ^c^autumn (September-November) precipitation; ^d^winter (December-February) precipitation; ^e^yearly precipitation; ^f^yearly net precipitation; ^g^yearly air temperature.

We tested the sensitivity of the HS6 δ^44/42^Ca values to severe drought events. The instrumental precipitation record in Yichang shows a sharp reduction of precipitation from 1896 to 1905 AD (Fig. 2). This severe drought event was also recorded in the 71-station averaged rainfall across China since 1880 AD^28^ and the dryness/wetness index^27^. Paralleling the severe drought recorded by the Yichang meteorological station, the HS6 δ^44/42^Ca values shows a positive excursion of near 0.2‰ (Fig. 2). Using the same one-box model as Owen *et al*.^14^, we obtain a mean precipitation of 980 mm during the severe drought event of 1896–1905 AD, which is higher than the 772 mm recorded in the instrumental record from the Yichang Station (Fig. 2). The overestimation of precipitation probably results from the accuracy of the simple one-box model. In addition, in years with quite low precipitation (e.g. 1896–1905 AD), the δ^44/42^Ca values in HS6 stalagmite are only a little higher than the mean values in HS4 from 8.5–7.9 ka. Such a difference may result from the quite different duration time of drought events; the 8.2-ka event lasted as long as 150 yr as recorded by the HS4 stalagmite^10^.

In the other stage with δ^44/42^Ca deviation different from the instrumental error (1961–1975 AD), the annual precipitation measured in Yichang does not show a persistent decrease, but it records droughts in single years. The changes of annual precipitation in Yichang City are in accord with the drought index smoothed over the two cities and the yearly precipitation anomaly in China (Fig. 2). However, the increase of δ^44/42^Ca values is a continuous pattern. In this case, we should be cautious with the different sensitivities between the δ^44/42^Ca values and the annual rainfall. The δ^44/42^Ca ratios may be more sensitive to drought events with duration time >10 yrs. This deduction is further supported by the correlation analysis based on 10-yr smoothed data, with improved correlation coefficients between δ^44/42^Ca and rainfall (Table 2). Such a pattern may result from the residence time of percolating water in Heshang Cave. Previous studies in this cave proposed that the retention time of percolating water is >1 yr^14,24,29^. Thus, under conditions when drought occurs in a single year or over less than 10 yr, the positive excursion of annual δ^44/42^Ca signals will be only moderate or even absent.

**Table 2:** Correlation analysis for Ca isotope values with precipitation, net precipitation, air temperature and the D/W index on the 10-yr smoothed data (^**^p < 0.01; ^*^p < 0.05; the abbreviations are identical with Table 1).

	δ^44/42^Ca	P_3–5_	P_6–8_	P_9–11_	P_12–2_	P_total_	P_eff_	Temp
P_3–5_^a^	−0.20							
P_6–8_^b^	−0.71^**^	0.36^**^						
P_9–11_^c^	0.33^**^	−0.24^*^	−0.10					
P_12–2_^d^	−0.13	0.33^**^	0.34^**^	−0.03				
P_tot_^e^	−0.48^**^	0.60^**^	0.86^**^	0.21	0.51^**^			
P_net_^f^	−0.69^**^	0.63^**^	0.90^**^	−0.14	0.37^**^	0.98^**^		
Temp^g^	−0.78^**^	0.51^**^	0.66^**^	−0.35^**^	0.39^**^	0.66^**^	0.57^**^	
D/W	0.67^**^	−0.46^**^	−0.86^**^	−0.13	−0.35^**^	−0.89^**^	−0.89^**^	−0.70^**^

**Figure 3:**
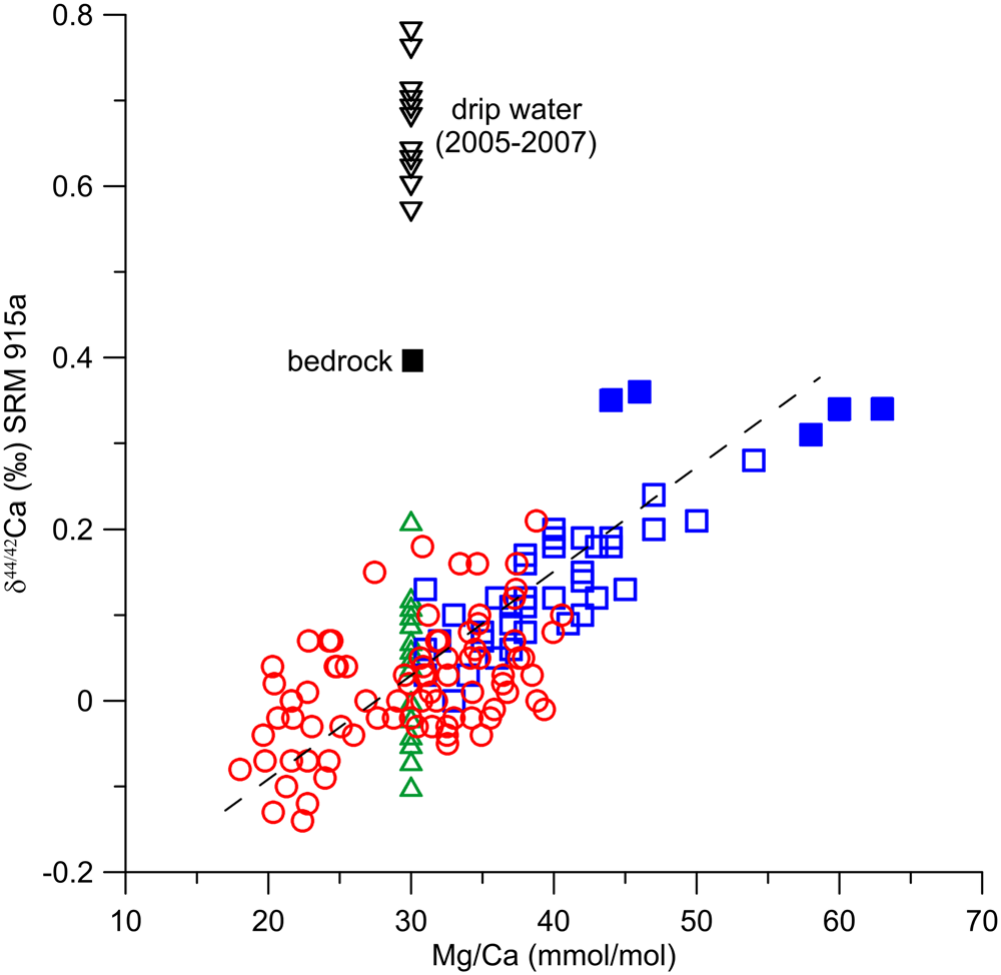
δ^44/42^Ca values comparisons among the HS6 stalagmite (red circle) over 1881–2001 AD with dripwater (black inverted triangle) and glass plate calcite (green triangle), and the HS4 stalagmite over 8.5–7.9 ka (blue square)^14^. For dripwater and glass plate calcite, the Mg/Ca values were set as 30 mmol/mol, which is the averaged value in HS6 stalagmite during 1881–2001 AD.

It is noteworthy that some data in HS6 stalagmite are very negative (<−0.10‰; Fig. 3). With the δ^44/42^Ca values of dolomite bedrock and the fractionation factor from Owen *et al*.^14^, if the PCP is completely absent, the deposited stalagmite δ^44/42^Ca values will be close to −0.2‰. Interestingly, the years with relatively negative δ^44/42^Ca values correspond with higher precipitation. It seems that enhanced precipitation may cause groundwater to flush quickly through the percolating layers until producing stalagmites.

### PCP reconstruction over 1881–2001 AD

The mean δ^44/42^Ca value (0.02‰) in HS6 from 1881–2001 AD is close to that of the mean values of glass plate calcite collected in the HS4 drip-site from 2005–2007 (0.05‰) and is negative relative to those in HS4 covering the 8.2 ka event (ca. 0.35‰) and in dripwater over a two-year monitoring experiment (0.68‰)^14^. These comparisons are consistent with the assumptions that dolomite bedrock (δ^44/42^Ca ≈ 0.40‰) is the primary Ca source for the HS4 and HS6 stalagmites and that PCP is an important process in controlling the Ca isotope compositions preserved in the Heshang Cave speleothems^14^.

In their study of Ca isotope compositions in Heshang Cave, Owen *et al*.^14^ established a simple model to estimate the fraction of Ca remaining in the solution:

1 $$f={(\frac{{r}_{s}}{a{r}_{0}})}^{\frac{1}{a-1}}$$

where *r*_s_ is the Ca isotope ratio of the instantaneous precipitation from dripwater (*r*_s_ = δ_CaCO3_/1000 + 1), *r*_0_ is the Ca isotope ratio of the initial dripwater, *α* is the Ca isotope fractionation factor between dripwater and calcite precipitation, and *f* is the proportion of Ca left in the solution. The fraction *f* is inversely closely related to PCP. When no PCP occurred, the *f* value is 1. In contrast, if almost all Ca is precipitated before reaching the cave ceiling, the *f* value is close to zero.

As done in Owen *et al*.^14^, the Ca isotope composition of dolomite bedrock was used as *r*_0_ (r_0_ = 1.0004 ± 0.00007), whereas the Ca isotope fractionation factor (*α* = 0.99937 ± 0.00003) between dripwater and glass plate calcite in modern conditions was used as the mean *α* for the period of 1881–2001 AD. In this short period, we can omit the influence of changes in the source compositions and in the Ca isotope fractionation factor. Thus, the *f* values can refer to the variations of PCP in the period 1881–2001 AD (Fig. 1).

The calculated *f* values vary between 0.50 and 0.86 (Fig. 1). The mean *f* value (0.68) in HS6 during 1881–2001 AD is a little higher than that over the 2005–2007 monitoring period (*f* = 0.64 ± 0.03; n = 15) and is relatively higher than the values covering the 8.2 ka event in the HS4 stalagmite (average 0.40)^14^. In the annual resolution PCP record from HS6 over the period 1881–2001 AD, three stages show a major decrease of *f* values (Fig. 1). All three stages are characterized by a decrease of ca. 0.10, which is half of the amplitude during the onset of the 8.2 ka event (0.20)^14^.

The influence of PCP on calcite precipitation is further supported by the elemental ratios of Mg/Ca, Sr/Ca and Ba/Ca, which also have the potential to record severe drought events and the associated PCP proportions^30^. In this study, the δ^44/42^Ca values correlate positively with Mg/Ca, but not with the other two elemental ratios (Table 3). During the interval with severe drought from 1896 to 1905 AD, both the Ca isotope composition and the Mg/Ca ratio show marked increases. The difference is that the increase of δ^44/42^Ca values happens in a brief period, whereas higher Mg/Ca ratios persist over a much longer time (Fig. 1). This comparison highlights that the δ^44/42^Ca values may be more sensitive to the changes of PCP proportions.

**Table 3:** Correlation analysis for Ca and O isotope compositions, Mg/Ca, Ba/Ca, and Sr/Ca (^**^p < 0.01; ^*^p < 0.05).

	δ^44/42^Ca	δ^18^O	Mg/Ca	Ba/Ca
δ^18^O	0.36^**^			
Mg/Ca	0.53^**^	0.01		
Ba/Ca	0.10	−0.37^**^	0.52^**^	
Sr/Ca	0.15	−0.34^**^	0.59^**^	0.81^**^

The elemental ratios of Mg/Ca, Ba/Ca, Sr/Ca change differently than the instrumental climate records (Table 3). The Mg/Ca ratio displays a negative relation with both the rainfall amount and air temperature, while the other two ratios have a very poor correlation with the climatic parameters. These comparisons with instrumental records highlight that these trace elemental ratios may be complicated by hydrochemical processes in the epikarst in addition to the water residence time. For example, in a recent study on the modern dripwater chemistry in the Hollow Ridge Cave, USA, Tremaine and Froelich^26^ concluded that dripwater Mg and Sr were controlled by mixing of sourced calcites and PCP. In a study of an annually layered stalagmite collected also in central China, the Sr/Ca and Ba/Ca ratio varied closely with the growth rate^31^.

## Conclusions

This study provides a near-annual resolution record of Ca isotope compositions from the HS6 stalagmite in central China with the aim to evaluate the potential to indicate aridity by directly comparing Ca isotope ratios with instrumental precipitation data. During the period from 1881 to 2001 AD, the δ^44/42^Ca values of the HS6 stalagmite ranged from −0.14‰ to 0.21‰. The correlation analysis clearly supports the hypothesis in Owen *et al*.^14^ that the stalagmite Ca isotope ratio is a powerful tool to infer past paleohydrological changes.

Using the model in Owen *et al*.^14^, the calculated PCP from Ca isotope compositions varied between 0.50 and 0.86, averaging 0.67. Two stages of time had with a markedly high PCP proportions: 1896–1905 AD and 1961–1975 AD. The first stage parallels a multi-year severe drought event recorded by the Yichang meteorological station. However, the latter stage of enhanced PCP proportions occurred in periods with drought only in single years. This inconsistency may result from the residence time of percolating water that is more than 1 yr in Heshang Cave and/or the influence of factors other than PCP. Thus, this study clearly shows that the δ^44/42^Ca values in the HS-6 stalagmite correlate more effectively with precipitation on decadal timescales than on annual timescales. More study is needed to fill the knowledge gap between stalagmite Ca isotope ratios and hydrological conditions.
